# Head-to-head comparison of two loop-mediated isothermal amplification (LAMP) kits for diagnosis of malaria in a non-endemic setting

**DOI:** 10.1186/s12936-023-04809-7

**Published:** 2023-12-13

**Authors:** Anna-Clara Ivarsson, Elin Fransén, Ioanna Broumou, Anna Färnert, Kristina E. M. Persson, Sara Karlsson Söbirk

**Affiliations:** 1 Clinical Microbiology, Infection Prevention and Control, Office for Medical Services, Region Skåne, Lund, Sweden; 2 Department of Medicine Solna, Karolinska Institute, Stockholm, Sweden; 3 Centre for Molecular Medicine, Stockholm, Sweden; 4 Department of Infectious Diseases, Karolinska University Hospital, Stockholm, Sweden; 5 Division of Clinical Chemistry and Pharmacology, Department of Laboratory Medicine, Lund University, Lund, Sweden; 6 Clinical Chemistry and Pharmacology, Laboratory Medicine, Office for Medical Services, Region Skåne, Lund, Sweden; 7 Division of Infection Medicine, Department of Clinical Sciences, Lund University, Lund, Sweden

**Keywords:** Malaria, LAMP, Loop-mediated isothermal amplification, Diagnosis

## Abstract

**Background:**

Light microscopy and rapid diagnostic tests (RDT) have long been the recommended diagnostic methods for malaria. However, in recent years, loop-mediated isothermal amplification (LAMP) techniques have been shown to offer superior performance, in particular concerning low-grade parasitaemia, by delivering higher sensitivity and specificity with low laboratory capacity requirements in little more than an hour. In this study, the diagnostic performance of two LAMP kits were assessed head-to-head, compared to highly sensitive quantitative real time PCR (qPCR), in a non-endemic setting.

**Methods:**

In this retrospective validation study two LAMP kits; Alethia^®^ Illumigene Malaria kit and HumaTurb Loopamp™ Malaria Pan Detection (PDT) kit, were evaluated head-to-head for detection of *Plasmodium*-DNA in 133 biobanked blood samples from suspected malaria cases at the Clinical Microbiology Laboratory of Region Skåne, Sweden to determine their diagnostic performance compared to qPCR.

**Results:**

Of the 133 samples tested, qPCR detected *Plasmodium* DNA in 41 samples (defined as true positives), and the two LAMP methods detected 41 and 37 of those, respectively. The results from the HumaTurb Loopamp™ Malaria PDT kit were in complete congruence with the qPCR, with a sensitivity of 100% (95% CI 91.40–100%) and specificity of 100% (95% CI 96.07–100%). The Alethia^®^ Illumigene Malaria kit had a sensitivity of 90.24% (95% CI 76.87–97.28) and a specificity of 95.65% (95% CI 89.24–98.80) as compared to qPCR.

**Conclusions:**

This head-to-head comparison showed higher performance indicators of the HumaTurb Loopamp™ Malaria PDT kit compared to the Alethia^®^ illumigene Malaria kit for detection of malaria.

**Supplementary Information:**

The online version contains supplementary material available at 10.1186/s12936-023-04809-7.

## Background

Management of patients with malaria relies on rapid and accurate diagnosis to secure prompt treatment. The diagnostic methods recommended for malaria diagnosis by the World Health Organization (WHO) continue to be light microscopy and immunochromatographic rapid diagnostic tests (RDT) [1]. While the sensitivity and specificity of RDTs are usually > 95% for the most virulent malaria species, *Plasmodium falciparum*, the sensitivity is lower for other *Plasmodium* species as well as for low parasite density infections [2–5]. The diagnostic sensitivity and specificity of light microscopy is highly dependent on the skills of the microscopist, something that can be hard to maintain 24/7 in non-endemic settings such as Sweden [6–8]. In recent years however, nucleic acid amplification test methods, such as loop mediated isothermal amplification (LAMP), have been tested increasingly in epidemiological studies as well as in clinical settings and have been proposed as a highly sensitive, cost-effective alternative in non-endemic high resource settings [7, 9–13].

In LAMP, the DNA-targets are amplified without the temperature cycles of the PCR-technique. Instead, a DNA-polymerase with strand displacement activity makes multiplication of the DNA-target possible at a constant temperature. The sequences targeted by the primers lead to the formation of DNA loops during amplification, which allows a highly sensitive and specific reaction and a shorter time to detection (time from starting the DNA-amplification reaction until a threshold is reached and interpreted as a positive result) than is usual for PCR. The DNA-amplification reaction forms a white precipitation of magnesium pyrophosphate, detected as turbidity or as fluorescence when exposed to UV light (if dyed with calcein). LAMP methods usually do not quantify the DNA content of the sample, which qPCR methods may through a Cq/CT-value [7, 14].

LAMP techniques for diagnosis of malaria have been reported to offer high diagnostic performance compared to RDT and microscopy, in particular concerning low grade-parasitaemia, and can deliver results with higher sensitivity (97–100%) and specificity (99.2 – 100%) with low laboratory capacity requirements in a little more than an hour [6–8, 11, 15–22]. The high diagnostic performance has been shown to include also non-*falciparum* species [18, 20, 23]. The above-mentioned challenges with current malaria diagnostics and the promising results of LAMP techniques have led to the question if LAMP could replace RDT and microscopy as a first line, point-of-care test for malaria in non-endemic high resource settings, such as Sweden. If LAMP could be used to confidently diagnose and rule out malaria in negative cases microscopy would only be needed in positive cases to determine *Plasmodium* species and parasite density. This study was designed to assess the performance of the two internationally available LAMP kits with a complete diagnostic solution for detection of *Plasmodium* DNA on the international market today: the Alethia^®^ Malaria Illumigene (Meridian Bioscience product code: 149,372) with reagents in the Malaria kit or Malaria PLUS kit, and the HumaTurb C + A or Humaloop M (Human Diagnostics Worldwide product codes: 963,200 or 962,000) with Loopamp™ which have several kits; Malaria Pan Detection kit and separate kits for species identification of *P. falciparum* or *Plasmodium vivax* (Table 1). The aim of this study was to compare these two LAMP instruments and kits by testing the Malaria kit for the Alethia® Malaria Illumigene instrument against the Loopamp™ Pan Detection (PDT) kit for the HumaTurb C + A instrument. Hereafter they will be referred to as Alethia® Illumigene Malaria kit and HumaTurb Loopamp™ Malaria PDT kit. These two kits have been compared to conventional methods in different settings but have not previously been compared head-to-head on the same samples [7, 12]. There are available diagnostic LAMP-kits for other pathogens, such as *Clostridium difficile, Mycobacterium tuberculosis, Mycobacterium pneumoniae,* but only the above mentioned kits for malaria have been compared in this study [24, 25].

**Table 1:** Characteristics of Alethia^®^ Illumigene Malaria kit and HumaTurb Loopamp™ Malaria PDT kit

	Alethia^®^ illumigene Malaria	HumaTurb Loopamp™ Malaria PDT
Mean time to result as measured in the study	44 min	60 min
Time to result as reported by the manufacturer^a^	< 45 min	50–55 min
Mean active time as measured in the study	4 min	10 min
Analysis^a^	Qualitative	Qualitative + turbidity graph and time to detection
Maximum samples per run^a^	10	16 (up to 94 if expanded with 6 HumaTurb A units)
Species identification^a^	Not possible	Not possible
Validated for species detection	*P. falciparum, P. vivax, P. ovale, P. malariae.* Performance for *P. knowlesi* established using purified genomic DNA only; whole organism testing has not been performed	*P. falciparum, P. vivax, P. ovale, P. malariae.* *P. knowlesi validated in one study* [20]^b^
Read out of the results^a^	Turbidity in Illumipro-10™ incubator or by eye	Turbidity in turbidimeter or fluorescence detected by eye
Limit of detection^a^	2.0 p/µL for *P*. *falciparum* and 0.1 p/µL for *P*. *vivax*	1 p/µL

^a^As described by the manufacturer [14, 30, 32]

^b^ [20]

This study was performed as a retrospective laboratory validation study of the analytic performance of two LAMP assays on biobanked samples from patients with suspected malaria in Region Skåne. The main objective was to investigate which of the two LAMP kits that had the best diagnostic performance compared to highly sensitive qPCR in patients with suspected imported malaria in a non-endemic setting at the Clinical Microbiology Department of Skåne in the South of Sweden. Another aim was to assess if any of the two LAMP kits could be used to generate a semiquantitative measurement of parasitaemia level.

## Methods

### Study design and study site

The analytical performance of two LAMP instruments and kits for detection of *Plasmodium*-DNA in biobanked blood samples were retrospectively evaluated at the Clinical Microbiology Laboratory of Region Skåne, Sweden to determine their performance compared to highly sensitive qPCR. Performance was defined as sensitivity and specificity compared to qPCR to detect DNA in the first diagnostic blood sample sent to the lab from patients with clinical suspicion of malaria.

The Clinical Microbiology Laboratory of Region Skåne is the main laboratory for the Skåne region in the south of Sweden, serving 9 hospitals and covering a population of 1.4 million. The positivity rate of suspected malaria samples at the Clinical Microbiology Laboratory of Region Skåne during the study time was 8–10% yearly (unpublished data from the Laboratory Information System, Region Skåne). The routine diagnostic method for malaria at the Clinical Microbiology Laboratory of Region Skåne at the time of the study was RDT and light microscopy. Blood samples were taken in EDTA-tubes from patients with suspected malaria and tested with the immunochromatographic rapid diagnostic test CareStart Malaria HRP2/pLDH (Pf/PAN) Combo Test at the point of care laboratory. In addition, microscopy of Giemsa-stained slides (irrespective of RDT result) was performed by an infectious disease specialist at the point of care laboratory, assessing *Plasmodium* species and parasitaemia. Various ID-specialists in four hospitals had the task to perform microscopy, and for many of them, this was a task rarely performed. Both RDT and microscopy were performed for all cases of suspected malaria. After diagnosis, whole blood samples (EDTA) were routinely sent to the regional Clinical Microbiology Laboratory without further processing and stored at − 80 °C in the Region Skåne biobank 136, Klinisk mikrobiologi BD1.

### Ethics statement

The study was approved by the Swedish Ethical Review Authority (Etikprövningsmyndigheten) ID 2020-05249.

### Study population and sample collection

Blood samples were selected among the biobanked samples previously sent to the Department of Clinical Microbiology on suspicion of malaria during the period 2018–2020. The blood samples that had once been reported as positive for *Plasmodium* spp. were identified through the laboratory system and selected for inclusion in the study. For every positive sample selected, the two consecutive samples that had been reported as negative for *Plasmodium* spp. were also included. Only one sample per patient was used. In total 51 positive samples and 102 negative samples were selected resulting in 153 included samples.

Exclusion criteria were cases for which samples were not identified in the bio bank, had insufficient volume for testing in the sample tube or inconclusive information about malaria diagnosis in the clinical files.

### Clinical data collection

Medical charts from included patients were obtained from Region Skåne medical databases. Epidemiological patient data was retrieved regarding sex, age, country of residence, country of origin, country of exposure, reason for travel, previous malaria episodes, chemoprophylaxis, time of exposure, time from symptom onset, malaria treatment initiated, presence of risk factors and comorbidities and data on parasitaemia and *Plasmodium* species from microscopy performed by clinicians. The above described patient data was collected to describe the study population from which samples were analysed in order to facilitate comparison with other settings outside of Sweden. The data is presented in Additional file 1.

### LAMP

Each of the included frozen whole blood samples obtained from the biobank were tested retrospectively with two LAMP-kits: Alethia^®^ Malaria illumigene Malaria kit (Meridian Bioscience product code: 480,925) and HumaTurb C + A Loopamp™ Malaria Pan Detection kit (Human Diagnostics Worldwide product code: 974,000). The tests were performed according to the manufacturer’s instructions after training by the supplier. The LAMP analysis of Alethia^®^ Illumigene Malaria kit is qualitative and is reported as positive, negative, or invalid. HumaTurb Loopamp™ Malaria PDT kit is a qualitative test that also reports time to detection. Neither of the two tested LAMP kits offer species differentiation. The LAMP analyses were performed by research personnel together with staff at the Clinical Microbiology Laboratory of Region Skåne during 2021 and 2022. Results from microscopy were blinded to the research personnel performing the LAMP assays. Invalid results were re-tested once and if the result remained invalid the sample was reported as invalid.

### Real time PCR

A total volume of 50 μL EDTA blood from each sample was used for genomic DNA extraction using the QIAamp DNA blood mini kit (Qiagen) and the protocol ‘DNA purification from Blood or Body fluids (spin protocol)’, according to manufacturer’s instructions. To reach a volume of 200 μL, starting material required for the specific extraction protocol, 150 μL of PBS 0.01 M pH 7.4 was added to 50 μL EDTA blood, according to manufacturer’s recommendation. At the final step, the genomic DNA was incubated for 5 min at room temperature prior to elution in 150 μL elution buffer and stored at −20 ℃.

For *Plasmodium* species detection and identification, a previously published multiplex, probe-based qPCR assay method was used [26], with the following modifications: 0.2 µM of primers Viv-F, Mal-F and Ova-F, 0.5 µM of primer Plasmo2-R and 0.1 µM of probes Falprobe, Vivprobe, Malaprobe, Ovaprobe (sequences available in Additional file 2). The cycling conditions were: 95 °C for 20 s, followed by 45 cycles at 95℃ for 15 s and at 60℃ for 1 min. Each sample was analysed in triplicates. Positive controls with tenfold serial dilutions of *P. falciparum, P. vivax, Plasmodium malariae* and *Plasmodium ovale* samples of known parasitaemia and non-template negative controls were used in each run. A cycle threshold (CT) value of 40 was used as a cut off to define positive samples. The limit of detection was 0.5–6 parasites for the different *Plasmodium* species. In case of inconsistent qPCR results, i.e., one out of three replicates positive, the qPCR was repeated, and the sample was analysed again in triplicate. If the result from the repeated qPCR matched the result from the first qPCR, the sample was considered as positive. Hence, one positive result out of three in the first run that was repeated in the second run was considered positive. If the repeated qPCR was negative the sample was considered negative.

### Statistical analysis

Sensitivity and specificity of LAMP techniques as compared to qPCR were calculated with the online statistical tool MedCalc [27]. The binominal test in IBM SPSS (version 29.0.1.0 (171) was used to calculate the if there was a significant difference in the sensitivity and specificity between the Alethia^®^ Illumigene Malaria kit and the HumaTurb Loopamp™ Malaria Pan Detection (PDT) kit. The non-parametric Spearman’s Rank Correlation test in IBM SPSS Statistics (version 29.0.1.0 (171)) was used to calculate all correlations; the two non-linear correlations between HumaTurb Loopamp™ Malaria PDT kit time to detection and qPCR CT value and parasitaemia as reported by microscopy as well as the linear correlation between qPCR CT value and HumaTurb Loopamp™ Malaria PDT kit time to detection.

## Results

**Fig. 1:**
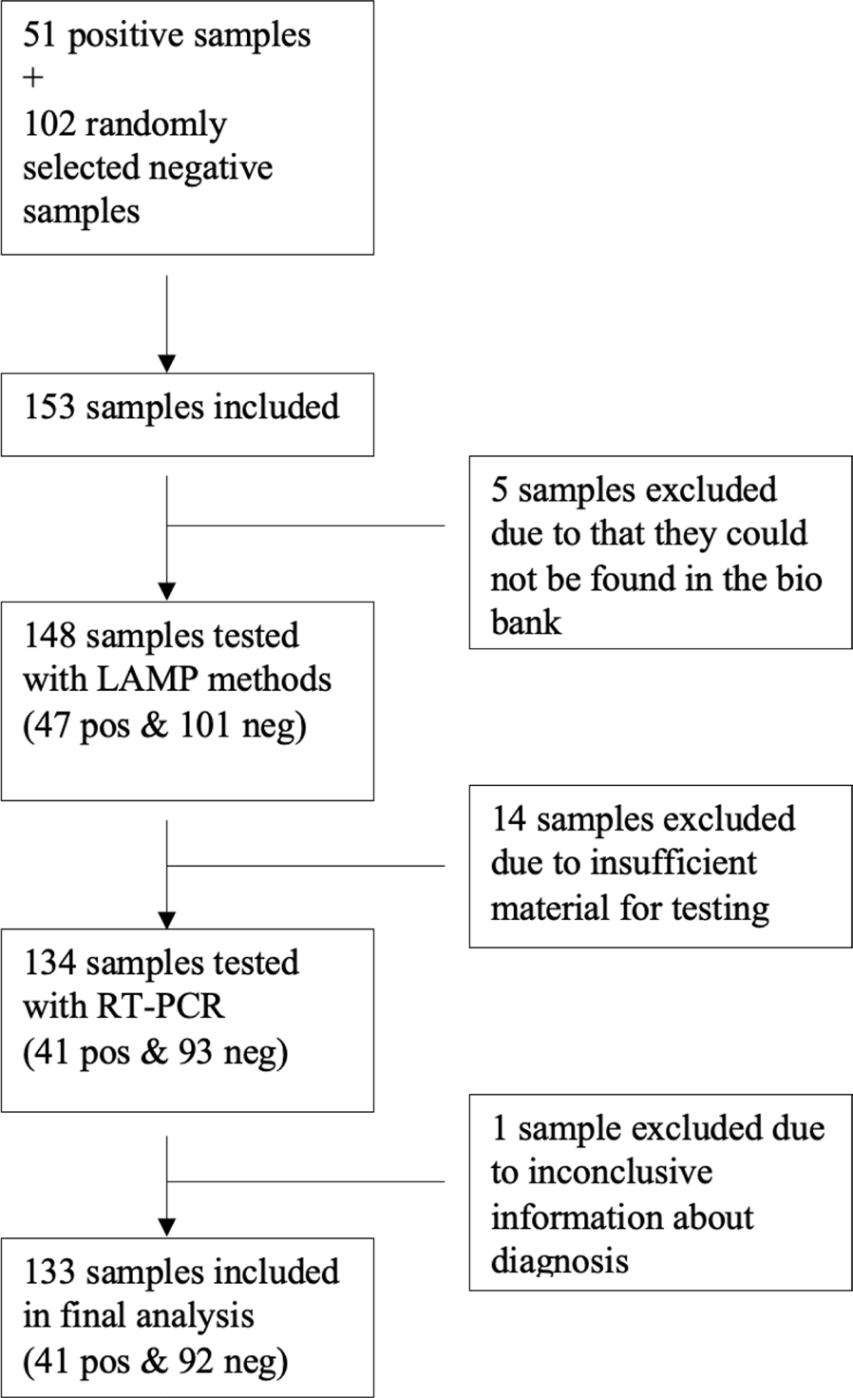
Sample selection flow chart

A total of 51 positive samples from patients diagnosed with malaria 2018–2020 were identified in the biobank list. In addition to these, 102 consecutive negative samples were selected resulting in a total of 153 samples. Of these, 5 samples could not be located in the biobank leaving a total of 148 samples that were tested with the two LAMP techniques, 47 previously registered as positive and 101 as negative. Of these samples, 14 had insufficient sample volume for qPCR, resulting in a total of 134 samples analysed by qPCR. The diagnosis of one of these samples was inconclusive with negative results in all diagnostic tests except one positive result out of six qPCR replicates (three primary replicates and three repeated replicates) with a high CT value of 38. The final clinical diagnosis of this patient was also unclear in the medical records. Therefore, this sample was excluded, resulting in a total of 133 samples analysed by both qPCR and the two LAMP kits. In total 41 samples were positive by qPCR and 92 were negative. The sample selection flowchart is detailed in Fig. 1.

The demographic characteristics of the 47 included positive cases that were tested with the two LAMP kits are presented in Additional file 1. Sex and age distributions are similar in cases and controls. Most cases were exposed in sub-Saharan Africa, visiting friends and family, and had not taken malaria chemoprophylaxis. The parasite densities (as measured through light microscopy by various clinicians) ranged between 0.1 and 4%.

### Diagnostic performance

Diagnostic performance of Alethia^®^ Illumigene Malaria kit and HumaTurb Loopamp™ Malaria PDT kit as compared to the reference method qPCR is presented in Tables 2 and 3. Previous results from microscopy and RDT is presented as additional information but was not part of the validation.

**Table 2:** Diagnostic performance of LAMP techniques compared to qPCR

Analysis	Sensitivity		Specificity	
	% (95% CI)	Proportion	% (95% CI)	Proportion
Alethia^®^ Illumigene Malaria	90.24% *(76.87–97.28)*	37/41	95.65% *(89.24–98.8)*	88/92
HumaTurb Loopamp™ Malaria PDT	100% *(91.40–100)*	41/41	100% *(96.07–100)*	92/92

**Table 3:** Malaria species in study samples tested positive vs. negative with LAMP techniques compared to qPCR

Total positive and species identified by qPCR	Study analyses			Previously performed diagnostics		
	qPCR	HumaTurb Loopamp™ Malaria PDT	Alethia^®^ Illumigene Malaria	Microscopy—correctly identified as pos. or neg.	Microscopy—correct species^a^	RDT -correctly identified as pos. or neg.
*Total positive*	41	41	37	39^b^	33	40
*P. falciparum*	31	31	28	29	25	30
*P. ovale*	4	4	4	4	4	4
*P. vivax*	5	5	4	5	4	5
*Mix P. falciparum *and* P. ovale*	1	1	1	1	0	1
*Negative samples*	92	92	88	92	–	91

^a^For some of the incorrect species’ identification by microscopy, one of the two species reported in the patient file was correct

^b^In one of the samples the patient had recently gone through successful treatment, and the microscopy is expected to be negative

The results from the qPCR and HumaTurb Loopamp™ Malaria PDT kit were in complete congruence, resulting in a sensitivity of 100% (95% CI 91.40–100%) and specificity of 100% (95% CI 96.07–100%) for the HumaTurb Loopamp™ Malaria PDT kit as compared to the qPCR. When comparing the Alethia^®^ Illumigene Malaria kit to qPCR there were 4 false positive and 4 false negative results leading to a sensitivity of 90.24 (95% CI 76.87–97.28) and a specificity of 95.65% (95% CI 89.24–98.80). The difference in sensitivity and specificity between the HumaTurb Loopamp™ Malaria PDT kit as compared to the Alethia^®^ Illumigene Malaria kit is statistically significant with a p-value of 0.015 and 0.017, respectively.

The Alethia^®^ Illumigene Malaria kit correctly identified 125 of 133 samples as either positive or negative (accuracy 94%) but failed to detect four cases of malaria and misdiagnosed four cases as positive. All four false positive results were negative in qPCR, microscopy and RDT. The clinical diagnosis for the four cases were viral infection or uncertain diagnosis. Out of the four false negative results two were positive in qPCR, microscopy and RDT with a noted parasite density of 0.2%. One of the cases had slightly inconclusive qPCR results with 2 out of 6 replicates (33.3%) positive in two independent qPCR experiments with a CT value of 40 in the first round and 38 in the second, negative microscopy and negative RDT. The clinical presentation of this case was a person with origin from an endemic area that was visiting friends and family and had had symptoms for one day. At presentation the case was diagnosed as non-malaria but three weeks later (without possible new exposure) malaria was diagnosed at another hospital. The last false negative case was positive in two of three qPCR replicates with a CT value of 40, positive in microscopy and positive in RDT. This case had used oral self-treatment before seeking care and was first interpreted as cured but was later reassessed as incompletely cured and put on malaria treatment with oral atovaquone/proguanil once more which led to clinical improvement.

The reference method qPCR identified *P. falciparum, P. ovale* and *P. vivax*, along with one mixed infection with *P. falciparum* and *P. ovale* in the study samples*.* Both LAMP assays correctly identified one or more samples with malaria caused by all above mentioned species as positive, although one *P. vivax* sample and three *P. falciparum* samples were missed by the Alethia^®^ Illumigene Malaria kit. The results (although it was not the aim of the study) show that species identification by microscopy had been a challenge in the current diagnostic setting with only 33 of 41 samples correctly identified by the microscopist as compared to the qPCR (Table 3). Two qPCR positive samples were also missed in microscopy and reported as microscopy negative, one of which was from a patient who had recently gone through successful treatment. In four samples out of the 148 analysed with the two LAMP techniques, the first result from the Alethia^®^ Illumigene Malaria kit was invalid. Upon retesting, all four samples had valid results. No invalid results were noted with the HumaTurb Loopamp™ Malaria PDT kit*.*

### Correlation between HumaTurb Loopamp™ Malaria PDT time to detection and qPCR CT value

**Fig. 2:**
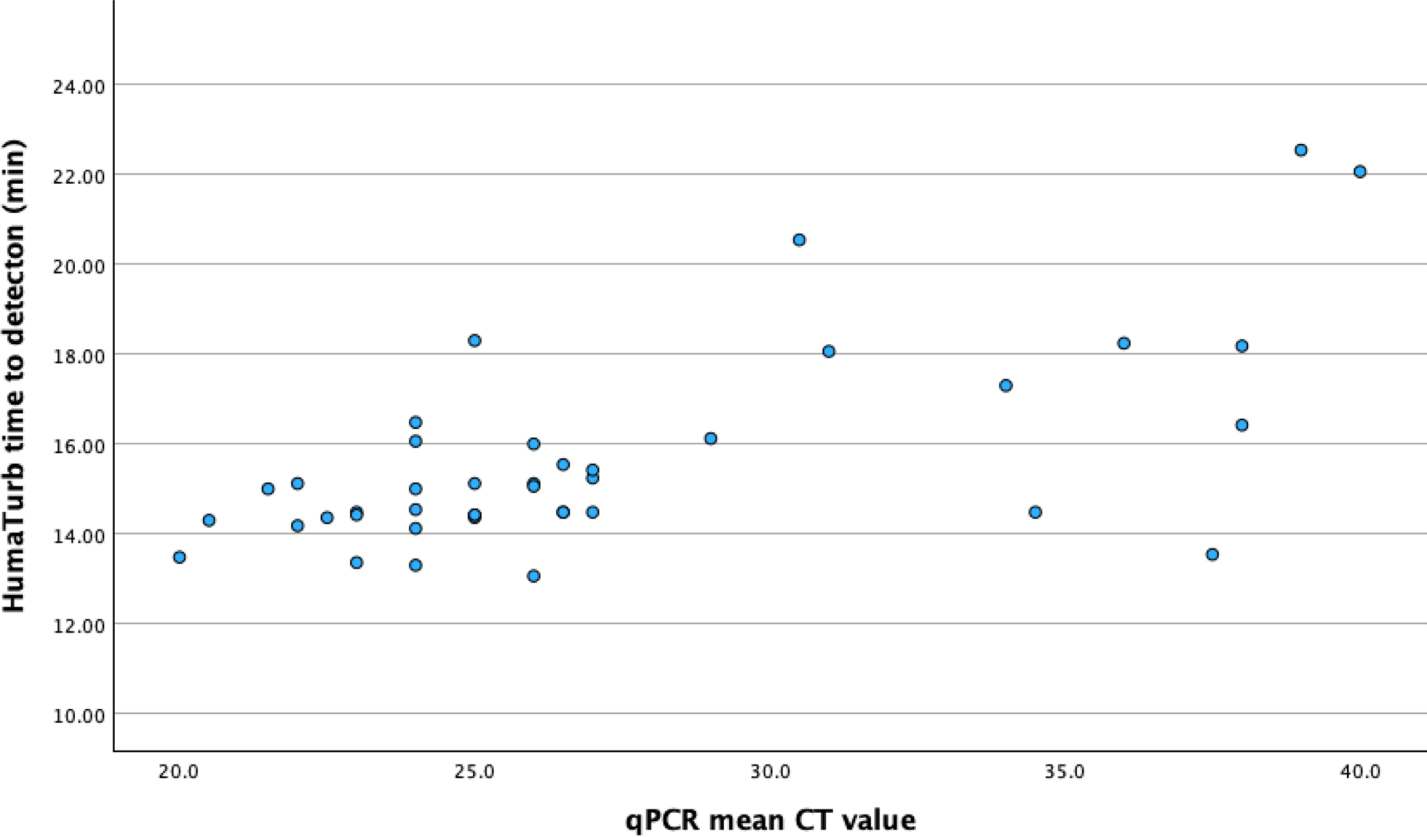
Correlation between HumaTurb Loopamp™ Malaria PDT time to detection and mean qPCR CT value

**Fig. 3:**
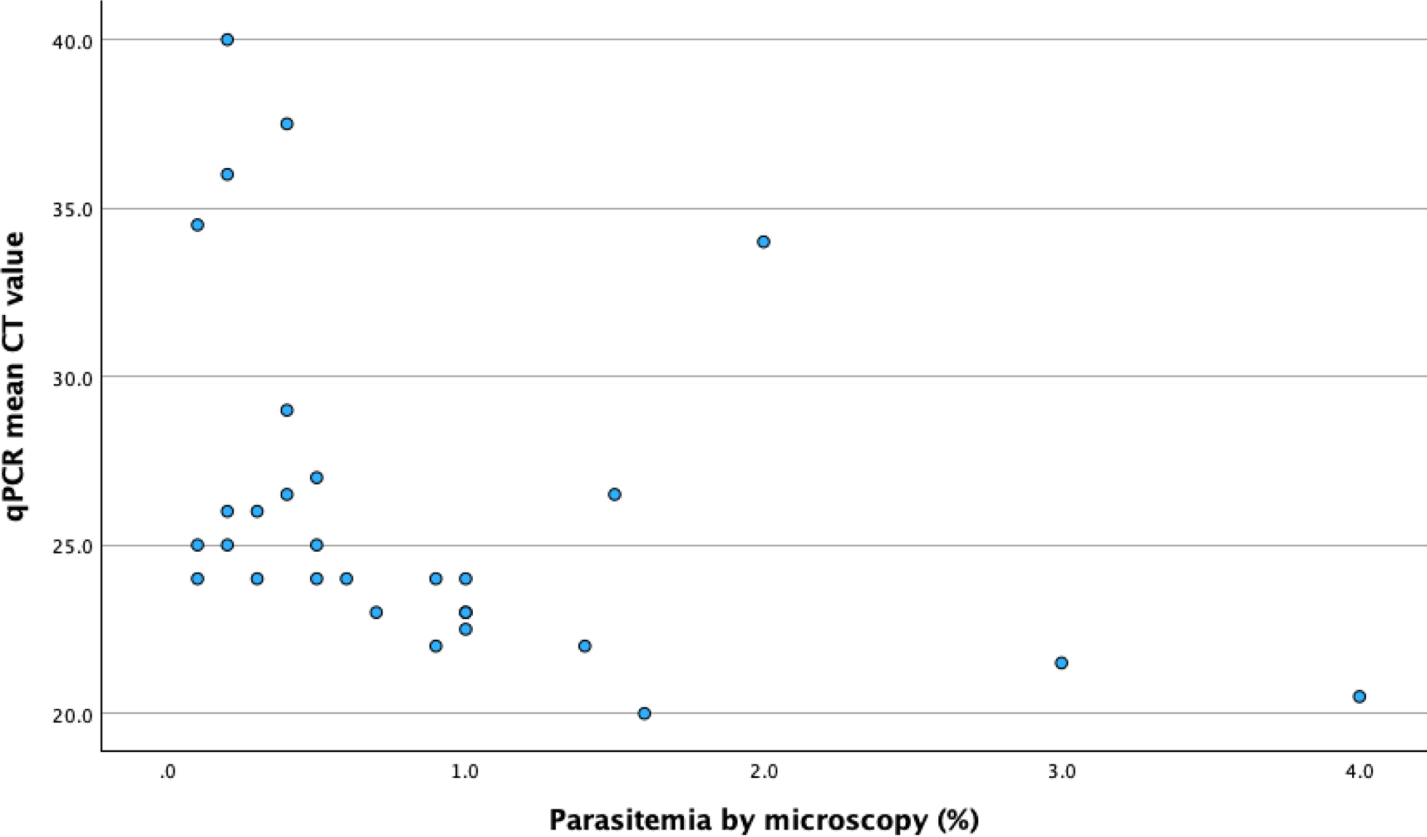
Correlation between mean qPCR CT value and parasitaemia

**Fig. 4:**
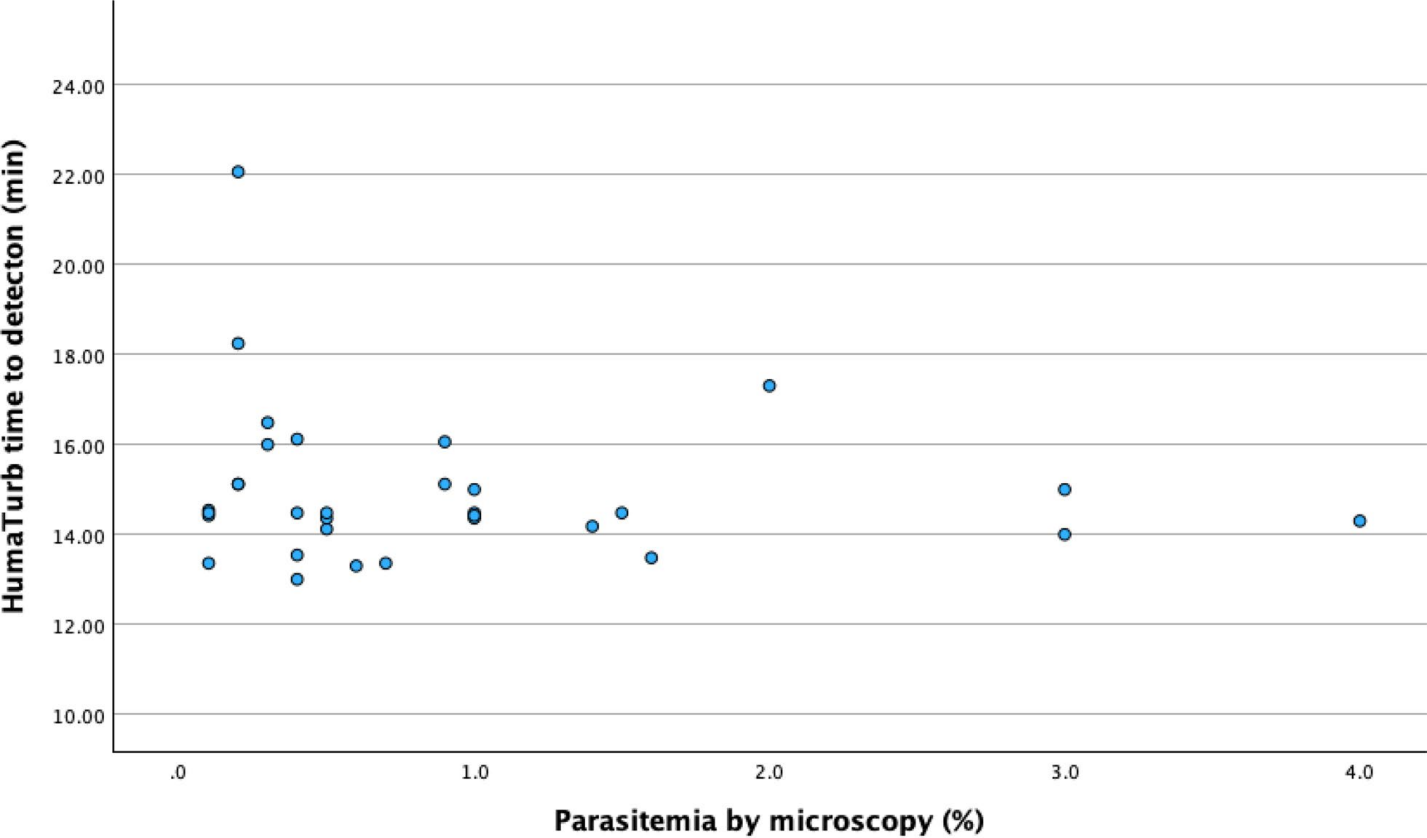
Correlation between HumaTurb Loopamp™ Malaria PDT time to detection and parasitaemia

There was a statistically significant positive correlation between the HumaTurb Loopamp™ Malaria PDT kit time to detection and the CT value of the qPCR. The correlation coefficient for the mean qPCR CT value in the three replicates and HumaTurb Loopamp™ Malaria PDT kit time to detection was 0.58 (p < 0.001) (Fig. 2). There was also a statistically significant negative correlation between mean qPCR CT value and level of parasitaemia (correlation coefficient − 0.601 (p < 0.001)) (Fig. 3). No statistically significant correlation could be seen between HumaTurb Loopamp™ Malaria PDT time to detection and level of parasitaemia as reported in the file at the time of diagnosis (correlation coefficient − 0.209, p = 0.251) (Fig. 4).

## Discussion

Management of patients with malaria relies on rapid and accurate diagnosis to secure prompt treatment, also in non-endemic settings, where travelers and migrants from endemic areas may present with symptoms. In this retrospective validation study the performance of two LAMP kits; Alethia^®^ illumigene Malaria kit and HumaTurb Loopamp™ Malaria Pan Detection (PDT) kit, was evaluated head-to-head for detection of *Plasmodium* DNA in 133 bio banked blood samples from suspected malaria cases at the Clinical Microbiology Laboratory of Region Skåne, Sweden compared to qPCR. The results show that the HumaTurb Loopamp™ Malaria PDT kit had a 100% sensitivity and specificity compared to highly sensitive qPCR. The Alethia^®^ illumigene Malaria kit correctly identified 125 of 133 samples as either positive or negative (accuracy 94%) but failed to detect four cases of malaria and misdiagnosed four samples as positive.

It is important to note that it is not possible to compare the diagnostic performance of RDT or microscopy versus the LAMP assays with this study design, since positive samples in this study were included on the basis of RDT and microscopy results which were the current laboratory methods for the malaria diagnosis in Skåne during the time of the study.

This data shows a significant positive correlation between qPCR CT value and time to detection with the HumaTurb Loopamp™ Malaria PDT kit. qPCR CT value has previously been shown to correlate with level of parasitaemia [28]. This is an area where further research could be beneficial, as levels of parasitaemia may be difficult to measure for inexperienced microscopists and thus alternative ways to estimate parasitaemia would be beneficial. The difficulties to measure level of parasitaemia might explain the weak correlation between reported level of parasitaemia and HumaTurb Loopamp™ Malaria PDT kit time to detection. A study comparing time to detection in the HumaTurb Loopamp™ Malaria PDT kit with level of parasitaemia by microscopy performed by an accredited laboratory with experienced staff would be of interest to evaluate the possible use of time to detection as an indirect semiquantitative measure of parasitaemia.

If the LAMP technique is to be used as a first diagnostic test to detect malaria, the 4 false negative results by the Alethia^®^ Illumigene Malaria kit are problematic. It is likely that two of the cases had very low levels of circulating *Plasmodium* DNA and parasitaemia, indicated by qPCR CT values close to the cut off (CT values 38 and 40), which could have been below the limit of detection for the Alethia^®^ Illumigene Malaria kit. In two of the samples, however, the parasite density was 0.2% (CT values 25 and 26) and no obvious reason for failure to detect *Plasmodium* DNA can be found. It is possible that the freezing of the samples for 2–4 years has damaged the parasite DNA, and thus leads to failure to detect the DNA, but this would most likely have been seen in the HumaTurb Loopamp™ Malaria PDT kit as well. Other possible reasons for false negative results could be the presence of inhibitors or the handling of the sample during the LAMP process.

A secondary finding is that species identification by microscopy was a challenge with only 33 of 41 positive samples previously correctly identified by the microscopist, and one case of malaria, which was positive in qPCR and one of the two LAMP techniques had been reported as negative in previous microscopy. The clinical reality in Sweden and many other non-endemic countries is that the microscopy is done at the point of care by the infectious disease doctor on call. The on-call doctor might perform malaria microscopy very rarely and might have had the microscopy training a very long time ago. This makes both sensitivity, species determination, and assessment of parasitaemia a challenge and may have affected a possible correlation between actual level of parasitaemia and time to detection by the HumaTurb Loopamp™ Malaria PDT kit. The problems associated with maintaining diagnostic skills adequately high for doctors on call outside office hours is one of the main reasons more sensitive methods are needed.

As previously mentioned, no other study comparing the performance of the HumaTurb Loopamp™ Malaria PDT kit and the Alethia^®^ Illumigene Malaria kit has been identified when searching the literature. The high sensitivity and specificity of the LAMP tests shown in this study are in line with what have been found in other similar studies. A large systematic review and meta-analysis based on 66 studies of diagnostic accuracy of the same two LAMP methods compared to PCR, microscopy and RDT for malaria diagnosis found a sensitivity and specificity > 0.95 of both methods in most of the included studies [29]. The reviewed studies did not include a head-to-head-comparison between the two different LAMP instruments. Another systematic review and meta-analysis based on 29 studies from Ethiopia also showed excellent sensitivity (100%) and specificity (86–99%) of LAMP compared to PCR [5].

## Strengths and limitations

A strength of this study is the comparison of two LAMP methods with highly sensitive qPCR head-to head in the same samples for which “field microscopy” and RDT at the point of care were known. However, a larger sample size would have given increased power to the statistical calculations.

This study is performed on samples that were frozen for 2–4 years which does not accurately represent the test material that will be used in practice, which might lead to unforeseen differences in diagnostic performance. However, all samples have been handled the same way and, according to the manufacturers, both kits may be used on frozen samples [14, 30]. The malaria positivity rate in this material is 31% which is very much higher than it will be in practical use at the labs in most non-endemic countries [6, 7, 22, 31]. The selection of samples by positive results in RDT and microscopy leads to very few low parasite density infections in the studied group, which might lead to an overestimation of the diagnostic accuracy of both LAMP instruments in this study. However, one case that presented with fever since one day, that was negative in RDT and microscopy and later was found to be malaria positive upon retesting, was diagnosed as positive by HumaTurb Loopamp™ Malaria PDT kit and qPCR. This could be a result of the higher sensitivity of molecular techniques (including LAMP) reported in previous studies, but the sample size is too small to draw any conclusions in the current study [7].

The laboratory analyses were performed by trained biomedical scientists as well as non-laboratory trained hospital staff that had gone through a short training, and it is possible that handling errors by non-laboratory trained staff may have contributed to contamination. Nonetheless, since these tests are planned to be run at the point-of-care it is important that non expert laboratory trained staff can run the tests with maintained accuracy and quality.

The cost-effectiveness has not been assessed in this study and is highly dependent on local factors, why it ideally should be done in each setting.

## Added value of this study

This study is the first study to compare the performance of the two leading LAMP instruments for diagnosis of malaria on the same material. The results show a high performance of both instruments but a superior performance of the HumaTurb Loopamp™ Malaria PDT kit.

## Conclusion

In this head-to-head retrospective validation study of the diagnostic performance of two LAMP kits compared to qPCR, the HumaTurb Loopamp™ Malaria PDT kit had a significantly higher diagnostic performance than the Alethia^®^ illumigene Malaria kit. Further studies with larger sample size to confirm these results would be beneficial. The high diagnostic sensitivity and specificity of HumaTurb Loopamp™ Malaria PDT kit along with ease of use and time to result around one hour makes this analysis suitable as a first line point of care diagnostic test for malaria in non-endemic high resource settings globally. However, the inability of these methods to perform full species differentiation and estimate the level of parasitaemia mean that they cannot yet completely replace microscopy in clinical diagnosis as this data is crucial for accurate treatment.

### Supplementary Information

**Additional file 1. **Demographic characteristics of positive cases.

**Additional file 2. **Sequences of used primers and probes for plasmodium species detection and discrimination.

## Data Availability

The datasets supporting the conclusions of this article are included within the article and its additional files.
