# Supplementary material for: Head-to-head comparison of two loop-mediated isothermal amplification (LAMP) kits for diagnosis of malaria in a non-endemic setting
Source: Malar J. 2023 Dec 13;22:377. doi: 10.1186/s12936-023-04809-7 (PMC10717323; doi:10.1186/s12936-023-04809-7)
Supplement: Supplementary file 1 — Additional file 1. Demographic characteristics of positive cases. [file 12936_2023_4809_MOESM1_ESM.docx]

Additional file 1 – Demographic characteristics of positive cases

| Characteristics of malaria cases (n=47) | n | *%* |
| --- | --- | --- |
| Sex |  |  |
| Men | 27 | *57%* |
| Women | 20 | *43%* |
| Age |  |  |
| Median age (range) | 36 y (6y-75y) |  |
| Region of infection |  |  |
| West Africa | 25 | *53%* |
| East Africa | 9 | *19%* |
| Central Africa | 7 | *15 %* |
| Central Asia | 3 | *6%* |
| South Asia | 3 | *6%* |
| Patient origin from endemic area |  |  |
| Yes | 35 | *74%* |
| No | 12 | *26%* |
| Reason for exposure |  |  |
| Visiting friends and family | 18 | *38%* |
| Migrant | 16 | *34%* |
| Tourist | 9 | *19%* |
| Residency in endemic area | 4 | *9%* |
| Previous malaria episodes |  |  |
| Yes | 27 | *57%* |
| No | 8 | *17%* |
| No information | 12 | *26%* |
| Chemoprophylaxis |  |  |
| Yes | 5 | *11%* |
| No | 39 | *83%* |
| Incomplete prophylaxis | 3 | *6%* |
| Parasitaemia % |  |  |
| 0.1 – 0.4 | 13 | *28%* |
| 0.5 – 0.9 | 10 | *21%* |
| 1 – 1.9 | 6 | *13%* |
| 2 – 2.9 | 2 | *4%* |
| 3 - 4 | 4 | *9%* |
| No data | 12 | *26%* |
