# Supplementary material for: Head-to-head comparison of two loop-mediated isothermal amplification (LAMP) kits for diagnosis of malaria in a non-endemic setting
Source: Malar J. 2023 Dec 13;22:377. doi: 10.1186/s12936-023-04809-7 (PMC10717323; doi:10.1186/s12936-023-04809-7)
Supplement: Supplementary file 2 — Additional file 2. Sequences of used primers and probes for plasmodium species detection and discrimination. [file 12936_2023_4809_MOESM2_ESM.docx]

Additional file 2: Sequences of used primers and probes for plasmodium species detection and discrimination

| Name of primers/ probes | Sequence of primers/ probes |
| --- | --- |
| Fal-F | 5’-CCG ACT AGG TGT TGG ATG AAA GTG TTA A-3’ |
| Viv-F | 5’-CCG ACT AGG CTT TGG ATG AAA GAT TTT A-3’ |
| Mal-F | 5’-CCG ACT AGG TGT TGG ATG ATA GAG TAA A-3’ |
| Ova-F | 5’-CCG ACT AGG TTT TGG ATG AAA GAT TTT T-3’ |
| Plasmo2-R | 5’-AAC CCA AAG ACT TTG ATT TCT CAT AA-3’ |
| Falprobe | 5’-ABY-AGC AAT CTA AAA GTC ACC TCG AAA GAT GAC T-QSY-3’ |
| Vivprobe | 5’-JUN-AGC AAT CTA AGA ATA AAC TCC GAA GAG AAA ATT CT-QSY-3’ |
| Malaprobe | 5’-6-FAM-CTA TCT AAA AGA AAC ACT CAT-MGBNFQ- 3’ |
| Ovaprobe | 5’-VIC-CGA AAG GAA TTT TCT TAT T- MGBNFQ- 3’ |
